# Supplementary material for: Exchange of water for sterol underlies sterol egress from a StARkin domain
Source: eLife. 2019 Dec 4;8:e53444. doi: 10.7554/eLife.53444 (PMC6940019; doi:10.7554/eLife.53444)
Supplement: Transparent reporting form [file elife-53444-transrepform.docx]

***eLife’s* transparent reporting form**

We encourage authors to provide detailed information *within their submission* to facilitate the interpretation and replication of experiments. Authors can upload supporting documentation to indicate the use of appropriate reporting guidelines for health-related research (see [EQUATOR Network](http://www.equator-network.org/%20)), life science research (see the [BioSharing Information Resource](https://biosharing.org/)), or the [ARRIVE guidelines](http://www.plosbiology.org/article/info:doi/10.1371/journal.pbio.1000412) for reporting work involving animal research. Where applicable, authors should refer to any relevant reporting standards documents in this form.

If you have any questions, please consult our Journal Policies and/or contact us: [editorial@elifesciences.org](mailto:editorial@elifesciences.org).

**Sample-size estimation**

- You should state whether an appropriate sample size was computed when the study was being designed
- You should state the statistical method of sample size computation and any required assumptions
- If no explicit power analysis was used, you should describe how you decided what sample (replicate) size (number) to use

Please outline where this information can be found within the submission (e.g., sections or figure legends), or explain why this information doesn’t apply to your submission:

The computational studies reported in the manuscript have been designed to achieve robust sampling of various stages of the cholesterol egress process. Thus, we carried out unbiased ensemble MD simulations (see Replicates section below) to enhance sampling of relevant dynamics in the system. As clearly described in the Results, out of 100 Stage-2 simulations the complete process of cholesterol release was observed in 5 trajectories. The remaining simulations sampled various stages of this process and as such were used to formulate a mechanistic hypothesis underlying the release process which was then tested and validated in functional experiments as described in the manuscript.

Quantitative potential of mean force (PMF) calculations were used to ensure that the various stages of the release process were sufficiently sampled in our unbiased ensemble MD simulations. The PMF as a function of the collective variable describing cholesterol release showed multiple minima which was consistent with relative sizes of populations of different states along the release process obtained from the analysis of unbiased MD simulations. This is described in detail in Results.

**Replicates**

- You should report how often each experiment was performed
- You should include a definition of biological versus technical replication
- The data obtained should be provided and sufficient information should be provided to indicate the number of independent biological and/or technical replicates
- If you encountered any outliers, you should describe how these were handled
- Criteria for exclusion/inclusion of data should be clearly stated
- High-throughput sequence data should be uploaded before submission, with a private link for reviewers provided (these are available from both GEO and ArrayExpress)

Please outline where this information can be found within the submission (e.g., sections or figure legends), or explain why this information doesn’t apply to your submission:

Computational experiments described in the manuscript were carried out in multiple replicates, as detailed in Methods and throughout the Results. Thus, for the unbiased ensemble MD simulations of the wild type Lam4S2, Stage-1 simulations were run in 10 independent replicates, whereas Stage-2 simulations were run in 100 independent replicates. Similarly, the ensemble simulations of the mutant constructs were carried out in 10 independent replicates. For each biological system, statistical independence of the replicates was ensured by resetting velocities of each atom at the beginning of each replicate of the ensemble. In Stage-1 simulations of the wild type protein, we have encountered a mode of Lam4S2 binding to the membrane that was classified as non-physiological artefact due to the fact that simulations considered only isolated Lam4S2 domain and therefore this mode of protein-lipid interaction was not pursued further. This is described in detail in the Results section.

Experiments with purified proteins were performed using both biological and technical replicates as appropriate. Specific information is provided in the corresponding figure legends.

**Statistical reporting**

- Statistical analysis methods should be described and justified
- Raw data should be presented in figures whenever informative to do so (typically when N per group is less than 10)
- For each experiment, you should identify the statistical tests used, exact values of N, definitions of center, methods of multiple test correction, and dispersion and precision measures (e.g., mean, median, SD, SEM, confidence intervals; and, for the major substantive results, a measure of effect size (e.g., Pearson's r, Cohen's d)
- Report exact p-values wherever possible alongside the summary statistics and 95% confidence intervals. These should be reported for all key questions and not only when the p-value is less than 0.05.

Please outline where this information can be found within the submission (e.g., sections or figure legends), or explain why this information doesn’t apply to your submission:

Convergence of the umbrella MD simulations was assessed by plotting probability distributions of the collective variables in different umbrella windows. The extent of overlap in the distribution plots suggests that the umbrella MD simulations are well-converged. To estimate error-bars on the PMF plot (Figure 4A), we followed the standard procedure outlined in WHAM software. Specifically, for each umbrella window a de-correlation time was first calculated and then error bars were constructed with Monte Carlo bootstrapping error analysis in the WHAM software on the decorrelated data points. This is explained in the Methods section.

(For large datasets, or papers with a very large number of statistical tests, you may upload a single table file with tests, Ns, etc., with reference to sections in the manuscript.)

**Group allocation**

- Indicate how samples were allocated into experimental groups (in the case of clinical studies, please specify allocation to treatment method); if randomization was used, please also state if restricted randomization was applied
- Indicate if masking was used during group allocation, data collection and/or data analysis

Please outline where this information can be found within the submission (e.g., sections or figure legends), or explain why this information doesn’t apply to your submission:

Not applicable - the nature of the work does not require allocation of samples into experimental groups.

**Additional data files (“source data”)**

- We encourage you to upload relevant additional data files, such as numerical data that are represented as a graph in a figure, or as a summary table
- Where provided, these should be in the most useful format, and they can be uploaded as “Source data” files linked to a main figure or table
- Include model definition files including the full list of parameters used
- Include code used for data analysis (e.g., R, MatLab)
- Avoid stating that data files are “available upon request”

Please indicate the figures or tables for which source data files have been provided:

Computational data sets are available for the main figures (Fig 1C, 2B, 4A and 4B).
